# Field Validation of a Magnetic Sensor to Monitor Borehole Deviation during Tunnel Excavation

**DOI:** 10.3390/ma11091511

**Published:** 2018-08-23

**Authors:** Fujian Tang, Jianzhou Yang, Hong-Nan Li, Fuqiang Liu, Ningbo Wang, Peng Jia, Yizheng Chen

**Affiliations:** 1 State Key Laboratory of Coastal and Offshore Engineering, School of Civil Engineering, Dalian University of Technology, Dalian 116024, China; hnli@dlut.edu.cn; 2 Henan Tianchi Pumped Storage Energy Corporation, Nanyang 474664, China; yangjzjsps@126.com (J.Y.); 13603778056@163.com (F.L.); wangningbo99@163.com (N.W.); 17703731783@163.com (P.J.); 3 Holcombe Department of Electrical and Computer Engineering, Clemson University, Clemson, SC 29634, USA

**Keywords:** magnetic sensor, pumped-storage hydroelectricity, tunnel excavation, borehole deviation

## Abstract

In this article, a magnetic sensor is proposed to monitor borehole deviation during tunnel excavation. It is made by piling four super-strong N42 NdFeB cylinder magnets and then encasing them in an aluminum alloy hollow cylinder. The distribution of the magnetic field produced by the magnetic sensor and its summation with the geomagnetic field (GMF) in a global coordinate system are derived based on the theory of magnetic fields. An algorithm is developed to localize the position of the magnetic sensor. The effect of the GMF variation on the effective monitoring range of the magnetic sensor is also studied numerically. Field validation tests are conducted at Jinzhai Pumped-Storage hydroelectric power station, during the excavation of an inclined tunnel in Anhui Province of China. Test results show that the algorithm and the magnetic sensor are used successfully to detect the deviation of the borehole with an estimated error of approximately 0.5 m. The errors are mainly from the measurement errors of the coordinates, of both the test and the measurement points. The effective monitoring range of the magnetic sensor is dependent on the direction of the magnetic sensor as well as the variation of the GMF.

## 1. Introduction

Water power is a renewable energy which is economically cheap, clean, reliable and abundant, compared with other conventional energy sources such as coal and petroleum. Nowadays, the electric energy supplied by the hydropower plant shares 22% of the total electric energy in China [1], and it functions as a regulator for load and frequency of the electricity power network [2]. Additionally, to reduce the variability of the entire electricity power network, many pumped storage hydroelectricity (PSH) stations have also been constructed.

**Figure 1:**
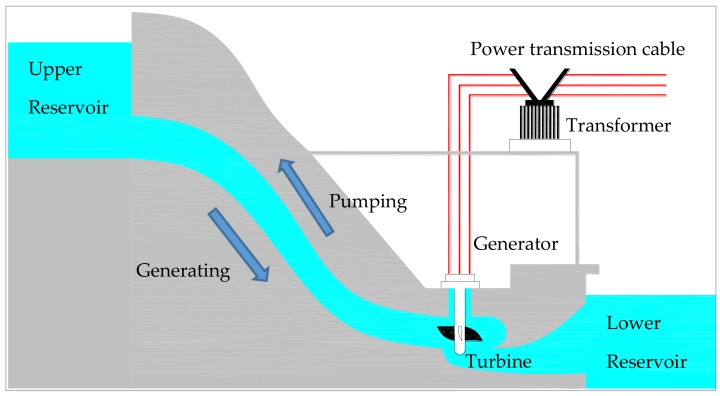
Schematic illustration of the pumped-storage hydroelectricity station.

The main function of PSH in the electricity power system works as a regulator for the variability of the power system, more specifically, to reduce the difference between the peak and the valley of the power network [3]. Figure 1 schematically illustrates the working mechanism of a PSH. During a period of low electricity demand, such as at night, the PSH pumps water from the lower reservoir to an upper reservoir, converting excessive electricity in the power network to potential energy of water. During high demand periods, like in the daytime, the PSH releases the water and the potential energy of water in the upper reservoir converts back to electricity, like a traditional hydroelectric power station. Therefore, the PSH system works as a peak-shaving and valley-filling of the electricity power network, spinning reserve capacity, phase modification and frequency control [3]. The efficiency of a PSH system is typically 70–80% [4,5].

Most of the PSHs are constructed in mountainous areas with great elevation differences. However, construction of tunnels for water pumping-generating cycling is usually a great challenge depending on the geotechnical conditions. Methods for tunnel excavation include a tunnel boring machine (TBM), cut-and-cover, and drilling and blasting (D&B). Although mechanical excavation with TBM is one of the most widely used methods for tunnel construction in the world [6], D&B is the most used tunnel construction method in China [7], since it is simple, cheap and not limited by geographical constraints. However, many safety issues often are involved with the D&B construction method, particularly for the excavation of inclined tunnels connecting horizontal tunnels at different elevations [8,9]. A commonly used construction method in China for the excavation of inclined tunnels includes three steps: First, a small inclined borehole with a diameter of around 30 cm is drilled through the mountain rock between the two horizontal tunnels; second, the diameter of the small hole is enlarged to a diameter of 2 m with a big drill bit; finally, the excavation of the inclined tunnel proceeds from the top to the bottom by using D&B method. There are at least two advantages for this type of tunnel excavation method. The excavated rocks and debris can be directly thrown into the big borehole, and dropped down to the bottom tunnel, then carried out by trucks. Some potentially damaged rocks or debris from previous blasting that had not dropped down could be removed, therefore, the safety of the construction workers can be guaranteed. 

Regarding long-distance drilling, directional control is always a challenge in many industries such as for oil and gas wells, coal mining, geothermal energy extraction and civil engineering. Borehole deviation is inevitable due to operational practices such as improper setup, excessive feed, misalignment, as well as geological conditions such as broken, incompetent ground, banded ground layers (successive soft and hard layers), the non-homogeneity of the rock, presence of rock fault, and so on [10,11]. When the deviation is great, the drill bit might be stuck inside the borehole and is hard to be removed, another borehole needs to be drilled again nearby. This is not only time-consuming, but also costly. It is estimated that in the USA alone, the drilling costs are billions of dollars annually in the petroleum industries [12]. 

A previous study proposed that a magnet-based smart rock could be used to monitor bridge scour effect, based on the principle of magnetic field interference [13]. The main purpose of the smart rock is to measure the scour depth of a bridge pier or foundation indirectly through the measurement of the coordinates of the smart rock. Via delivering the smart rock to a bridge pier or foundation, the scour depth can be determined by comparing the change in the coordinate of the smart rock during a flood event. 

During this study, a magnetic sensor, which is similar to smart rock technology, was proposed, and used to monitor the borehole deviation during tunnel excavation. The magnetic sensor is actually a large cylindrical magnet, which was made by piling four super-strong cylinder magnets, and then encasing them in an aluminum alloy hollow cylinder. It is taken to a PSH station for validation tests. An algorithm is developed to localize the position of the magnetic sensor based on the theory of magnetic fields and the minimization of a proposed objective error function. Moreover, the effect of the variation of the geomagnetic field on the effective monitoring range of the magnetic sensor also is studied numerically. 

## 2. Working Principle of Magnetic Sensor

The magnetic sensor developed in this study is actually a super-strong cylindrical magnet which is made by piling four N42 NdFeB permanent magnets together. Monitoring of borehole deviation during tunnel excavation is realized by delivering the magnetic sensor to the bottom of a borehole and continuously tracing its position during excavation through a megnetometer and localization algorithm. Therefore, this section deals with the development of a localization algorithm based on the theory of magnetic fields. 

### 2.1. Magnetic Field Produced by a Cylinder Magnet

**Figure 2:**
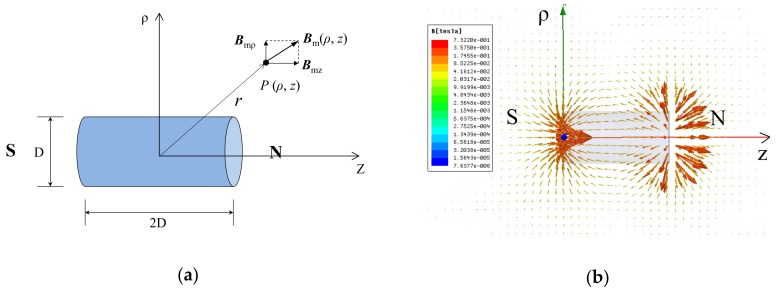
(**a**) The magnetic field vector of a cylinder magnet in a cylindrical coordinate system; (**b**) distribution of the magnetic field produced by a cylinder magnet. S, south pole; N, north pole; ρ, ρ-axis; Z, z-axis.

Figure 2a shows a cylinder magnet with D in diameter and 2D in height in a cylindrical coordinate system. The origin is located at the centroid of the magnet, z-axis is the direction from south (S) to the north (N) pole, and ρ-axis is the radial direction perpendicular to z-axis.

The magnetic field produced by the cylinder magnet is axially symmetrical around the z-axis and can be represented by a vector *B*_m_ (*ρ*, *z*) at a point *P* (*ρ*, *z*) as shown in Figure 2a. As point *P* is significantly farther from the cylinder magnet, the magnet can be modeled as a magnetic dipole. The corresponding magnetic flux density at point *P* is a highly nonlinear function of the position and direction of the cylinder magnet and can be decomposed into a longitudinal component *B*_mz_ and a radial component *B*_mρ_. The magnitudes of *B*_mz_ and *B*_mρ_ can be expressed as [14]:

(1) $$\left\{ \begin{array}{l} B_{mz,}(\rho ,z)=\;\frac{\mu_{0}\mu }{4\pi }\frac{\left(2z^{2}-\rho^{2}\right)}{r^{5}}\\ B_{m\rho }(\rho ,z)=\frac{\mu_{0}\mu }{4\pi }\frac{3\rho z}{r^{5}} \end{array}\right.,$$

where $\mu_{0}$ is the permeability of vacuum in T·m/A, and μ is the magnetic moment of the cylinder magnet. Therefore, the magnitude of the magnetic field intensity produced by the cylinder magnet at point *P* (*ρ*, *z*) is:

(2) $$B_{m}(\rho ,z)=\sqrt{B_{mz}^{2}(\rho ,z)+B_{m\rho }^{2}(\rho ,z)}.$$

The distribution of the magnetic field produced by a cylinder magnet was numerically simulated and shown in Figure 2b, in which the arrow is the direction of the magnetic field and the length represents the magnitude. It can be seen that the magnetic field comes out of the N pole and flows back to the S pole. The magnitude is not uniformly distributed (strong near the two ends, and weak near the side) and decreases rapidly as a function of the distance away from it. 

**Figure 3:**
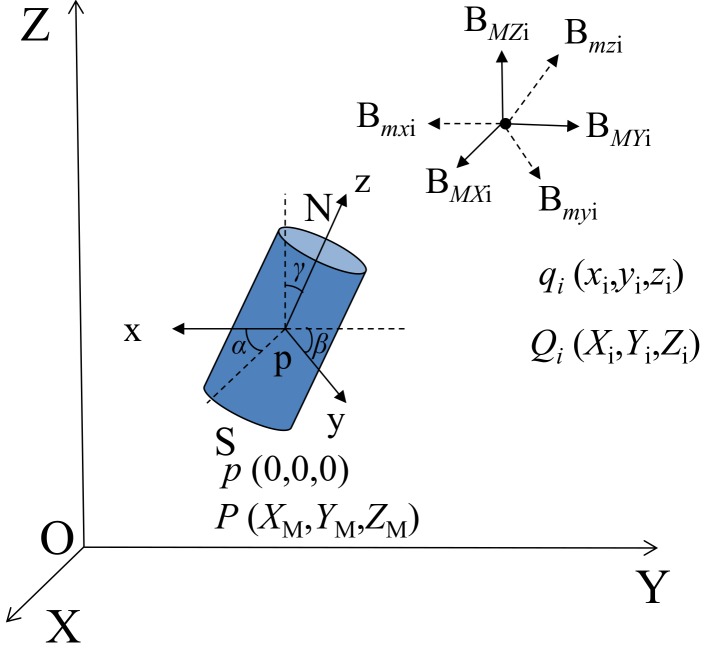
Schematic illustration of coordinate transfer.

The magnetic field induced by the cylinder magnet, as shown in Figure 2, is derived in a local cylindrical coordinate system. To be used for field applications, it needs to be transferred into a global Cartesian coordinate system at a test site. To ensure convenience of transition, a local Cartesian coordinate system is introduced. Figure 3 schematically illustrates the transition of the coordinate systems, in which a local Cartesian coordinate system p-xyz is in a global Cartesian coordinate system O-XYZ. Figure 3 shows the center point *p* (0, 0, 0) of the cylinder magnet is the origin of the local Cartesian coordinate system p-xyz, in which the z-axis is pointing from the south (S) to the north (N) pole and the x- and y-axis are in a plane perpendicular to the z-axis. Therefore, the origin *p* (0, 0, 0) of the local coordinate system corresponds to point *P* (*X*_M_, *Y*_M_, *Z*_M_) in the global coordinate system, and an arbitrary point *Q_i_* has a local coordinate *q_i_* (*x_i_*, *y_i_*, *z_i_*) and a global coordinate *Q_i_* (*X_i_*, *Y_i_*, *Z_i_*). 

Using a local Cartesian coordinate system, the intensity vector of a magnetic field at point *q_i_(x_i_*, *y_i_*, *z_i_)* can be expressed as *B_mi_* = (*B_mxi_*, *B_myi_*, *B_mzi_*). Its two components in a cylindrical coordinate system are given in Equation (1), in which the radial component can be further decomposed into x- and y-components. Therefore, the x-, y- and z-components of the magnetic intensity at point *q_i_* in a local coordinate system are:

(3) $$\left\{ \begin{array}{l} B_{mxi}=\frac{\mu_{0}\mu }{4\pi }\frac{3x_{i}z_{i}}{r_{i}^{5}}\\ B_{myi}=\frac{\mu_{0}\mu }{4\pi }\frac{3y_{i}z_{i}}{r_{i}^{5}}\\ B_{mzi}=\frac{\mu_{0}\mu }{4\pi }\frac{2z_{i}{}^{2}-x_{i}{}^{2}-y_{i}{}^{2}}{r_{i}^{5}} \end{array}\right.,$$

where

(4) $$r_{i}=\sqrt{x_{i}^{2}+y_{i}^{2}+z_{i}^{2}}.$$

To transfer from the local coordinate system to the global coordinate system, a transition matrix **R** is introduced [13]:

(5) $$(\begin{array}{l} x_{i}\\ y_{i}\\ z_{i} \end{array}),=R\left(\begin{array}{l} X_{i}-X_{M}\\ Y_{i}-Y_{M}\\ Z_{i}-Z_{M} \end{array}\right),$$

(6) $$R=[\begin{array}{ccc} \cos\beta \cos\gamma & \cos\beta \sin\gamma & -\sin\beta \\ \sin\alpha \sin\beta \cos\gamma -\cos\alpha \sin\gamma & \sin\alpha \sin\beta \sin\gamma +\cos\alpha \cos\gamma & \sin\alpha \cos\beta \\ \cos\alpha \sin\beta \cos\gamma +\sin\alpha \sin\gamma & \cos\alpha \sin\beta \sin\gamma -\sin\alpha \cos\gamma & \cos\alpha \cos\beta \end{array}],$$

where α∈[0,2π], β∈[0,2π], γ∈[0,2π] are the rotation angles of the x-, y-, and z-axis of the local coordinate system with respect to the X-, Y- and X-axis of the global coordinate system, as shown in Figure 3. 

Therefore, the three-components of the magnetic field intensity at any point *Q_i_* (*X_i_*, *Y_i_*, *Z_i_*) in the global coordinate system takes the form:

(7) $$\left(\begin{array}{l} B_{MXi}\\ B_{MYi}\\ B_{MZi} \end{array}\right)=R^{-1}\left(\begin{array}{l} B_{mxi}\\ B_{myi}\\ B_{mzi} \end{array}\right)=R^{-1}\left(\begin{array}{c} \frac{\mu_{0}\mu }{4\pi }\frac{3x_{i}z_{i}}{r_{i}^{5}}\\ \frac{\mu_{0}\mu }{4\pi }\frac{3y_{i}z_{i}}{r_{i}^{5}}\\ \frac{\mu_{0}\mu }{4\pi }\frac{2z_{i}{}^{2}-x_{i}{}^{2}-y_{i}{}^{2}}{r_{i}^{5}} \end{array}\right).$$

### 2.2. Determination of the Geomagnetic Field and the Total Magnetic Field in Global Coordinate System

**Figure 4:**
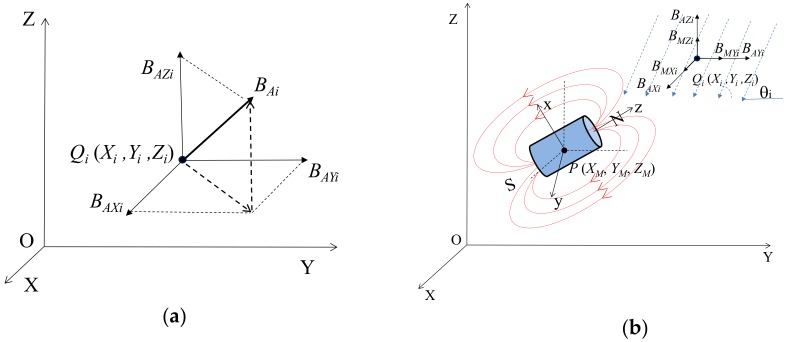
(**a**) Vector description of geomagnetic field (GMF); (**b**) summation of GMF and the magnetic field produced by the magnetic sensor in a global coordinate system.

Found in most cases, the magnetic field present at a test site is actually the geomagnetic field (GMF), which is also a vector in a global coordinate system, as shown in Figure 4a, and can be expressed as:

*B_Ai_* = *B_AXi_ i* + *B_AYi_ j* + *B_AZi_ k*, (8)

where (*B_AXi_*, *B_AYi_*, *B_AZi_*) are the three-components of the GMF, which can be determined by using a magnetometer. 

The magnetic field at a measurement point is a vector summation of the GMF and the magnetic field produced by the magnetic sensor, which is schematically illustrated in a global O-XYZ Cartesian coordinate system in Figure 4b. The cylindrical magnetic sensor is centered at point *P* (*X*_M_, *Y*_M_, *Z*_M_), and the inclination angle of the GMF at a point *Q_i_* (*X*_i_, *Y*_i_, *Z*_i_) is *θ*_i_. Therefore, the magnitude of the summation of the GMF and the magnetic field produced by the magnetic sensor *B_Ti_* = *B_Ai_* + *B_Mi_* at a point *Q_i_* (*X*_i_, *Y*_i_, *Z*_i_) can be expressed as:

(9) $$B_{Ti}=\sqrt{{\left(B_{MXi}+B_{AXi}\right)}^{2}+{\left(B_{MYi}+B_{AYi}\right)}^{2}+{\left(B_{MZi}+B_{AZi}\right)}^{2}},$$

where (*B_MXi_*, *B_MYi_*, *B_MZi_*) and (*B_AXi_*, *B_AYi_*, *B_AZi_*) are the three components of the magnetic field produced by the magnetic sensor, and the GMF at the test site, which are expressed in Equations (6) and (7), respectively. Usually, the GMF at a test site during a field test is considered to be constant. Therefore, the magnetic field at a measurement point is a function of the coordinate (*X*_M_, *Y*_M_, *Z*_M_) and the direction (related to angles of α, β and γ) of the magnetic sensor in the global coordinate system of the test site.

### 2.3. Localization Algorithm

The magnitude of the GMF *B_Ai_* and the total magnetic field *B_Ti_*, after delivering the magnetic sensor to a measurement point, can be measured by using a magnetometer. The magnitude of the magnetic field produced by the magnetic sensor itself also can be estimated theoretically based on the coordinate of the measurement point, which can be determined with a total station. Therefore, the location of the magnetic sensor can be obtained by minimizing the difference between the estimated *B_Ti_*^(*E*)^ and the measured *B_Ti_*^(*M*)^ values. Therefore, an objective error function to localize the location of the magnetic sensor is formulated as expressed in Equation (10), in which (*X*_M_, *Y*_M_, *Z*_M_) is the coordinate of the magnetic sensor in the global coordinate system of the test site.

It is assumed that there are *n* measurements of the magnitude of the magnetic field, *B_Ti_*^(*M*)^ (*i* = 1, 2, …, *n*), at *n* measurement points around the magnetic sensor. At each point, the theoretically estimated intensity of the total magnetic field from the GMF and the magnetic sensor *B_Ti_*^(*E*)^ = *B_Ti_* can be calculated by substituting Equations (7) and (8) into Equation (9). The root-sum-squares (RSS) error between the estimated intensity *B_Ti_^(E)^* and the measured intensity *B_Ti_^(M)^*, which is *J*(*X*_M_, *Y*_M_, *Z*_M_), can be expressed as:

(10) $$J(X_{M},Y_{M},Z_{M})=\sqrt{\sum_{i=1\;}^{n}{\left[B_{Ti}^{\left(E\right)}-B_{Ti}^{\left(M\right)}\right]}^{2}}.$$

Via minimizing the RSS error function in Equation (10), using the sequential quadratic programming (SQP) algorithm [15], the coordinate (*X*_M_, *Y*_M_, *Z*_M_) of the magnetic sensor can be determined. 

## 3. Field Validation Tests 

### 3.1. Jinzhai Pumped-Storage Hydroelectricity Station

**Figure 5:**
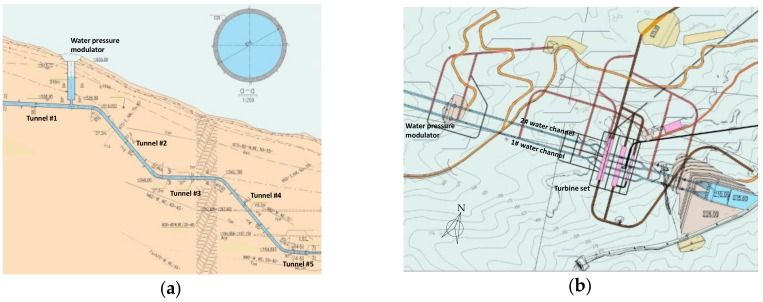
(**a**) Elevation view and (**b**) plan view of Jinzhai pumped storage hydroelectricity station.

**Figure 6:**
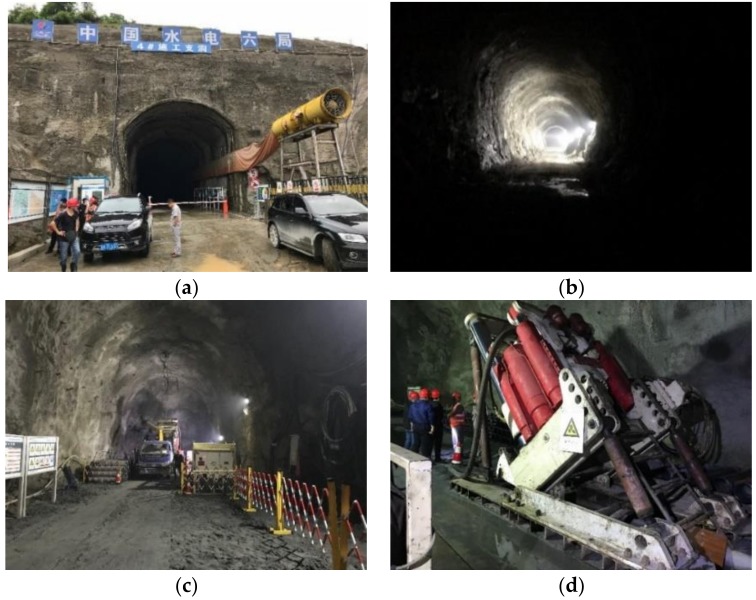
(**a**) Outside view of the construction tunnel; (**b**) inside the tunnel; (**c**) the end of top horizontal tunnel #1; (**d**) set-up of drilling machine for borehole in inclined tunnel #2.

Figure 5 shows the cross-sectional elevation view and the plan view of Jinzhai pumped-storage hydroelectricity station, which is located in Jinzhai County, in the west of Anhui Province, China. It was constructed in the Taihang Mountains, and the total elevation difference between the upper reservoir and the lower reservoir is over 400 m. Two parallel water tunnels were constructed, as shown in Figure 6b, and their diameters decrease gradually from 8.2 m in the top tunnel to 6.2 m in the bottom tunnel. The water tunnel consists of a total of five portions, as shown in the elevation view in Figure 6a: Three horizontal tunnels (tunnel #1, #3, and #5) and two inclined tunnels (tunnel #2 and #4) that connect the horizontal tunnels. Supplemental to the tunnels for water channels, there were a few construction tunnels used to transport the excavated rocks outside and construction materials inside, as shown in Figure 5b. Drilling and blasting was the construction method for tunnel excavation in this project. During the excavation of the top inclined tunnel, a 30 cm-diameter borehole was first drilled to connect tunnel #1 and tunnel #3. The borehole had a length of approximately 162 m and it deviated approximately 2.50 m from the center line (C.L.) at its bottom. Therefore, the borehole deviation for the excavation of inclined tunnel #2 was selected for field validation tests of the magnetic sensor.

Figure 6 shows the pictures of the construction tunnel for the water channels and the drilling machine of the borehole for tunnel #2. Figure 6a,b show the outside and the inside of one of the tunnels during excavation. Figure 6c,d show the end of top tunnel #1 and the set-up of the drilling machine for the borehole in the inclined tunnel #2. 

### 3.2. Fabrication of Magnetic Sensor

**Figure 7:**
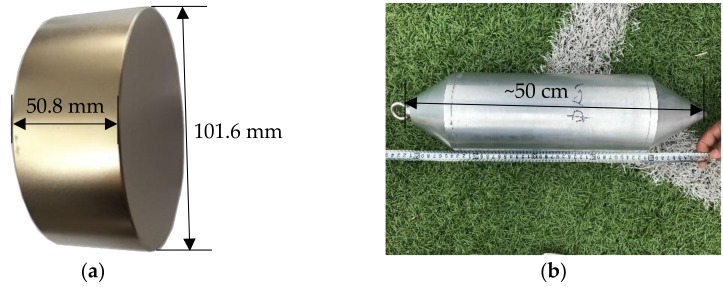
(**a**) N42 cylinder magnet, and (**b**) magnetic sensor fabricated in this study.

Figure 7a shows the cylinder magnet used to make the magnetic sensor in this study. It is a N42 NdFeB magnet with a diameter of 101.6 mm and a height of 50.8 mm. To increase the effective monitoring range of this magnetic sensor, four cylinder magnets were piled together to produce a super-strong cylindrical magnet with a diameter of 101.6 mm and a height of 203.2 mm. To protect the magnets from friction during delivery to the designated measurement points inside the borehole, an aluminum alloy hollow cylinder shell, with an outside diameter of 15 cm and a length of ~50 cm, was made as shown in Figure 7b. The piled cylinder magnets were encased in the aluminum alloy cylinder and an aluminum alloy ring also was welded at one end to connect with high-strength stainless steel tendons for delivery to designated measurement points during validation tests. 

### 3.3. Measurement of Magnetic Field Intensity

**Figure 8:**
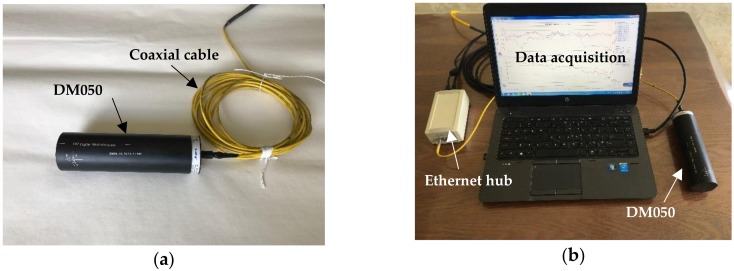
Magnetometer used for magnetic field measurement: (**a**) Magnetometer DM050, and (**b**) data acquisition system.

A digital 3-axis magnetometer system, as shown in Figure 8, manufactured by STL System technik Ludwig GmBH in Konstanz, Germany, was used for intensity measurement of the magnetic field. It is composed of a digital sensor DM050, a three-channel Ethernet hub, a 50 m long coaxial cable for power and data transmission, and a notebook with STL GradMag software installed for full control of measurement, data acquisition and graphical display. The DM050 is a precision magnetometer with 0.002 nT resolution, less than 0.06 $\mathrm{nT}/\sqrt{\mathrm{Hz}}$ noise and a field range of ±1 mT. It measures three orthogonal field components of a magnetic field at a maximum sample rate of 10 kHz. The software also offers the total magnetic field as an extra virtual channel. Typical sources of errors due to axis misalignment, scaling, offset and phase are eliminated to the greatest extent possible with a digital signal conditioning strategy. The software, offering full control over all system features, real-time monitoring of data and data documentation, greatly improves the efficiency of field data analysis and display.

### 3.4. Test Setup and Layout of Measurement Points

**Figure 9:**
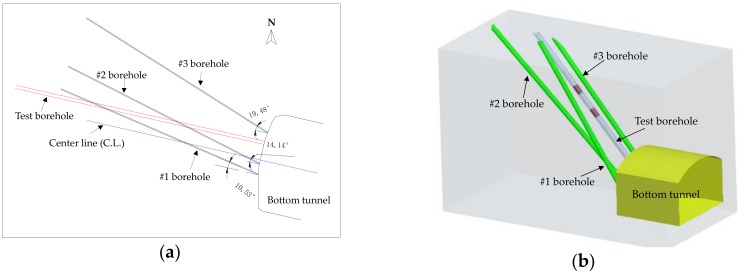
(**a**) 2-dimensional layout of all four boreholes; (**b**) 3-dimensional spatial demonstration of the test borehole and the three measurement boreholes (the test borehole is for tunnel #2 excavation and the other three boreholes #1, #2, and #3 are for deployment of measurement points).

Figure 9 shows the layout of the test borehole and three small measurement boreholes at the bottom end of tunnel #2 in a 2- and 3-dimensional demonstration. It can be seen that the test borehole deviated away 2.5 m from the centerline of the bottom tunnel, as shown in Figure 9a. To accurately localize the position of the bottom of the borehole, three small measurement boreholes were drilled inside the end rock wall of tunnel #3 around the test borehole and their horizontal angles respective to the centerline of the bottom tunnel #3 are also indicated, as shown in Figure 9a. Figure 9b shows the relative spatial locations of the three small measurement boreholes and the big test borehole. 

**Figure 10:**
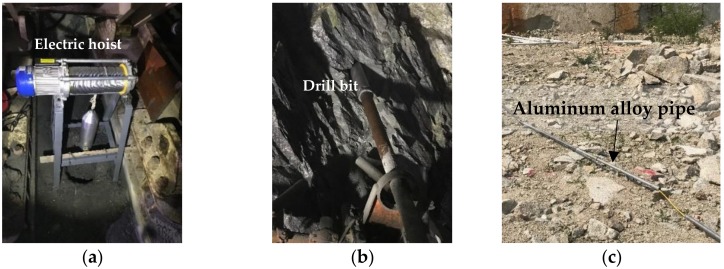
(**a**) Set-up of electric hoist to deliver the magnetic sensor to the bottom of the test borehole; (**b**) small borehole drilling for measurement points; (**c**) aluminum alloy pipe to deliver magnetometer to designated measurement points inside the small boreholes.

**Figure 11:**
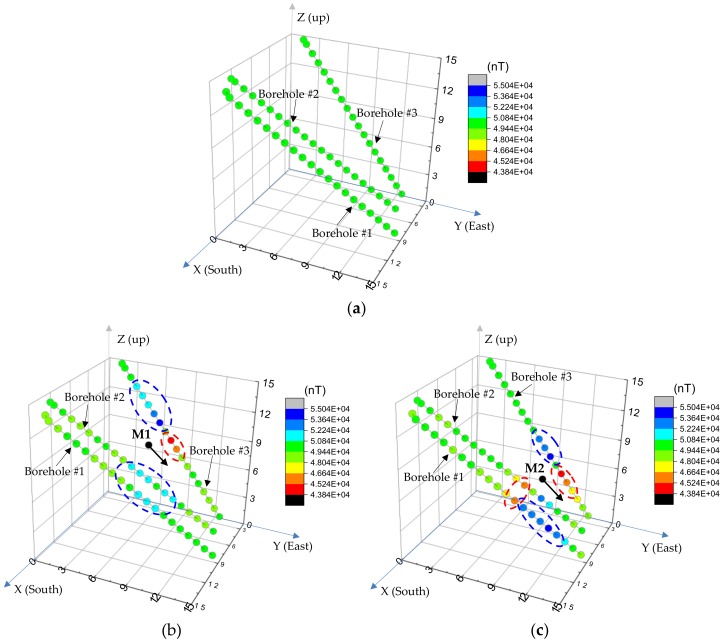
Distribution of the magnetic field intensity of (**a**) GMF; (**b**) the total magnetic field as the magnetic sensor was placed at location M1; (**c**) the total magnetic field as the magnetic sensor was placed at location M2 (unit: m).

Figure 10a shows the assembly to deliver the magnetic sensor to the bottom of the test borehole by using an electric hoist. Figure 10b shows the drill set-up for the three small measurement boreholes to deliver the magnetometer to measurement points inside them. The diameter of the small borehole was 100 mm with a length of 25 m. Measurement points were selected inside the three small boreholes with a spacing of ~1 m, as shown in Figure 11. To deliver the magnetometer to designated measurement points inside the boreholes, a total of 25 pieces of aluminum alloy pipes with a diameter of 101 mm and a length of 1.2 m were designed and fabricated, as shown in Figure 10c. The magnetometer was fixed at the top of the aluminum alloy pip, and aluminum alloy pipes were connected one-by-one to push the magnetometer to the designated measure points inside the borehole. A total of 61 measurement points were selected, including 19 in borehole #1, 21 in borehole #2 and 21 in borehole #3, as shown in Figure 11.

To execute the field tests, a total of three rounds of tests were performed. First, the magnitude of the geomagnetic field at these measurement points were measured with the magnetometer; next, the magnetic sensor was delivered to a position 10 m away from the bottom of the test borehole, and the magnitude of the total magnetic field at all the measurement points was recorded; last, the magnetic sensor was delivered to another position 6 m from the end of test borehole, and the magnitude of the total magnetic field was recorded again. During the tests, a total station was set up to measure the global coordinates of the measurement points. The coordinates of the measurement points inside the boreholes were determined by extrapolating the coordinates of two points outside the boreholes along its centerline. To determine the coordinates of the two points outside the boreholes, a 5 m long PVC pipe was inserted into the boreholes, and two points were marked on the PVC pipe and their coordinates were recorded by the total station. The measurement of the magnetic field intensity at all measurement points was conducted by using the magnetometer with a sampling rate of 5 Hz and a duration of 7 s. The average of the measurements was used for further analysis. 

### 3.5. Test Results and Discussion 

Figure 11 shows the distribution of the measured magnitude of the GMF at 61 measurement points inside the three small boreholes and their corresponding total magnetic fields, after the magnetic sensor was delivered to position M1 and M2 inside the test borehole, which was 10 m and 6 m away from the end of the bottom tunnel, respectively. A global coordinate system, O-XYZ, was set as shown in Figure 11, in which the Z-axis is pointing upwards, the X-axis is pointing south of the Earth, and the Y-axis is pointing east of the Earth. Every single dot corresponds to the spatial location of a measurement point in the global coordinate system and the color of the dot represents the magnitude of the magnetic field. 

Figure 11a shows the magnitude of the GMF at all the measurement points inside the three small boreholes is constant and has an average of 49,416.41 nT. Subsequent to delivering the magnetic sensor to location M1 (7.995, 7.298, 9.783), which was 10 m away from the end of the test borehole as shown in Figure 11b, the magnitude of the GMF around the magnetic sensor changed due to its interference with the magnetic field produced by the magnetic sensor. It is noted that for some measurement points in borehole #3, which were higher than M1 in the test borehole, the magnitude of the total magnetic field increased (blue color). Light blue dots are also present at other measurement points lower than M1 in borehole #2 and borehole #1. Conversely, the magnitude of the magnetic field decreased at points in borehole #3, which were lower than M1 (red color). This is because the distribution of the magnetic field produced by the magnetic sensor was not uniform and the direction and magnitude of the magnetic field varied point by point. Therefore, interference with the GMF resulted in an increase or decrease in the magnitude of the total magnetic field. 

When the magnetic sensor was delivered to a lower location M2 (7.809, 9.853, 6.712) in the test borehole, as shown in Figure 11c, changes in the magnitude of the GMF around the magnetic sensor also were observed, which was similar to that of the magnetic sensor at location M1. Comparing Figure 11c with Figure 11b, it can be observed that a darker color (red or blue) was present when the magnetic sensor was located at M2, than the dot color when the magnetic sensor was located at M1. Specifically, when the magnetic sensor was located at M2 a significant increase or decrease in the GMF was observed at measurement points surrounding it. This is because the measurement points surrounding location M1 were much closer to it than those surrounding location M2, resulting in a significant change in the GMF. The closer the measurement points were to the magnetic sensor, the more significant the interference to the GMF. 

The positions M1 and M2 of the magnetic sensor were calculated by using the localization algorithm and they were compared with the measured locations, as shown in Table 1. The measured coordinates of the two locations were extrapolated from two points outside the test borehole by using a total station. The estimated error was evaluated by using the root-sum-of-squares (RSS) principle as follows:

(11) $$\mathrm{RSS}=\sqrt{{\left(X_{i}^{E}-X_{i}^{M}\right)}^{2}+{\left(Y_{i}^{E}-Y_{i}^{M}\right)}^{2}+{\left(Z_{i}^{E}-Z_{i}^{M}\right)}^{2}},$$

where $\left(X_{i}^{E},Y_{i}^{E},Z_{i}^{E}\right)$ and $\left(X_{i}^{M},Y_{i}^{M},Z_{i}^{M}\right)$ are the estimated and measured coordinates of the test locations for M1 and M2, respectively.

It can be seen from Table 1 that RSS errors of the coordinates between the estimated and the measured were 0.490 m and 0.561 m as the magnetic sensor was placed at location M1 and M2, respectively. The source of error is mainly from the measurement error of the coordinate by extrapolation method. Firstly, the diameter of the magnetic sensor was 150 mm, which was greater than the diameter of the PVC pipe that is 80 mm, resulting in a misalignment of the centerline of the PVC pipe with the magnetic sensor. Secondly, the length of the PVC pipe used was 5 m, which actually did not represent the centerline of the test borehole. Therefore, these factors caused an error to calculate the coordinates of the magnetic sensor by extrapolation. Similarly, extrapolation from the two points outside the borehole also caused an error to the coordinates of the 61 measurement points inside the three small boreholes, particularly for the measurement points deep inside. However, the magnetic sensor technology developed in this study is still good to monitor the borehole deviation, and an error of ~0.50 m satisfies the engineering requirement. 

## 4. Effective Monitoring Range of Magnetic Sensor

The magnetic sensor for borehole deviation monitoring proposed in this study is based on the interference of the GMF after introducing the magnetic sensor. Through monitoring and comparing the change of the GMF before and after introducing a magnetic sensor, the location of the sensor can be determined. Due to the effect of the dynamo action in the upper atmosphere and the solar wind, the GMF varies over time. Moreover, the intensity of the magnetic field produced by a permanent magnet decays in a cubic function with a distance away from it, as shown in Figure 2b. Therefore, it is necessary to quantify an effective measurement zone within which the position of the magnetic sensor can be localized. Specifically, the change of the magnetic field intensity before and after delivery of the magnetic sensor can be recognized by the magnetometer. The effective monitoring principle is defined as:

(12) $$\left|B_{T}-\mu_{\mathrm{GMF}\;}\right|\geq 3\sigma_{\mathrm{GMF}}\;,$$

where *µ*_GMF_ and *σ*_GMF_ are the mean value and standard deviation of the GMF at the test site, respectively; *B*_T_ is the magnitude of the total magnetic field from the GMF and the magnetic sensor. 

The direction of the magnetic sensor was dependent on the inclination of the test borehole. Three representative directions were considered to investigate their effect on the effective monitoring zone, for simplicity in this study. The magnetic sensor used for numerical simulation was the magnetic sensor used for field tests, and the GMF was the GMF at the test site, which was measured, and had an intensity of 49,416.41 ± 33 nT and an inclined angle of 46.692°.

**Figure 12:**
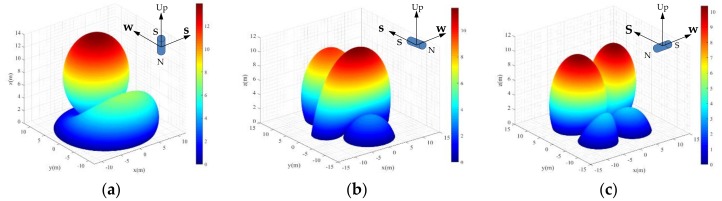
Effect of GFM variation on the effective monitoring range with standard deviation 33 nT as the direction of the magnetic sensor points (**a**) up/down; (**b**) south/north; (**c**) west/east.

Figure 12 shows the effective monitoring range of the magnetic sensor when its direction was pointing upwards/downwards (Figure 12a), south/north (Figure 12b), and west/east (Figure 12c) of the Earth. The effective monitoring ranges were the same as the direction of the magnetic sensor was pointing upwards/downwards, south/north, and west/east, since Equation (12) took the absolute value. Shown in these figures, the magnetic sensor was placed at the origin of the coordinate system and the color represents the height of the effective monitoring zone. To localize the position of the magnetic sensor, all the measurement points should be selected within this zone. It is noted that the effective monitoring zone was asymmetrical and dependent on the direction of the sensor. This is because, on one hand, the distribution of the magnetic field induced by a cylinder magnet was not symmetric, as demonstrated in Figure 2b. On the other hand, the GMF had an inclination angle of 46.692°. Therefore, these two factors resulted in either an increase or decrease of the magnitude of the total magnetic field. The maximum effective monitoring range in the vertical direction was around 14 m, 12 m, and 10 m as the S pole of the magnetic sensor was pointing up, south, and west of the Earth, respectively. 

## 5. Conclusions

During this study, a magnetic sensor was proposed, fabricated, and used to monitor the borehole deviation during tunnel excavation. The magnetic sensor was made by piling four super-strong N42 NdFeB cylinder magnets. An algorithm to localize the position of the magnetic sensor was developed based on the theory of magnetic fields. Field validation tests were conducted at Jinzhai pumped-storage hydroelectricity station in Anhui Province, China. Validation test results showed that the magnetic sensor and the localization algorithm can be used to effectively monitor the deviation of the borehole with an error of ~0.5 m. The error was mainly from measurement errors of the coordinates of both the test points and the measurement points by using extrapolation. The effective monitoring range of this magnetic sensor was dependent on the direction of the magnetic sensor, as well as the variation of the GMF. When the standard deviation of GMF was 33 nT, the maximum effective monitoring range in the vertical direction was 14 m, 12 m, and 10 m when the S pole of the magnetic sensor pointed up, south, and west of the Earth, respectively.

## Figures and Tables

**Table 1:** Estimated and measured locations of the smart rock.

Sensor Location	Estimated Coordinate			Measured Coordinate			RSS Error (m)
	*X*^E^ (m)	*Y*^E^ (m)	*Z*^E^ (m)	*X*^M^ (m)	*Y*^M^ (m)	*Z*^M^ (m)	
M1 (10 m)	8.104	7.769	9.704	7.995	7.298	9.783	0.490
M2 (6 m)	7.651	10.299	6.409	7.809	9.853	6.712	0.561
